# Oxidation of cysteine-rich proteins during gel electrophoresis

**DOI:** 10.14440/jbm.2018.275

**Published:** 2018-12-05

**Authors:** Cesare Achilli, Annarita Ciana, Giampaolo Minetti

**Affiliations:** Biochemical laboratories, Department of Biology and Biotechnology, University of Pavia, Pavia 27100, Italy

The first electrophoretic analysis of proteins was performed in 1937 by Arne Tiselius [1], who was awarded the Nobel Prize in Chemistry 1948 for this important contribution. This fundamental analytical technique, which had a decisive impact on research in such disciplines as biochemistry, microbiology, immunology and molecular biology, has been perfected over the years. In present days, the most common type of electrophoretic separation of proteins is based on their preventive denaturation by sodium dodecyl sulfate (SDS). This amphipathic detergent binds to the denatured proteins in a well calibrated ratio, abolishes their original intrinsic net charge and confers to them a negative charge of uniform density. Proteins so treated are separated by polyacrylamide gel electrophoresis in SDS (SDS-PAGE). In the gel, they all migrate towards the anode, with an electrophoretic mobility correlated with their mass. Discontinuous SDS-PAGE was then introduced, by Leonard Ornstein and Baruch J. Davis in 1964 [2,3], as a more efficient variant of SDS-PAGE. It consists of a stacking gel polymerized on top of the resolving gel. The stacking gel, by its more acidic pH and lower polyacrylamide concentration, allows proteins to stack into a thin band before entering into the resolving gel, where they separate as better resolved bands.

Concerning the visualization of proteins in gel, two main strategies have taken hold: the non-specific protein staining with dyes such as coomassie brilliant blue [4], or the specific detection of a given protein by Western blotting [5]. In addition, an SDS-PAGE variant, later to be named zymography, was introduced for in-gel visualization of proteins endowed with hydrolytic activity, especially proteases. This method is based on incorporation into the polyacrylamide gel of a specific substrate for the protease under investigation. After electrophoretic separation, the gel is incubated in a suitable buffer to ensure that the proteases possibly present in the original sample acquire again their enzymatic activity and digest the substrate *in situ*. The gel is then stained, for instance with coomassie blue, and the sites of proteolysis appear as white bands on a blue background [6].

SDS-PAGE, as a method for separating proteins according to their mass, has been further improved by Ulrich K. Laemmli in 1970 [7]. In the new protocol the protein samples are denatured with SDS in the presence of 2-mercaptoethanol, a reducing agent that cleaves any disulfide bond, whether native or artificially induced, between cysteine residues in proteins. The compound also prevents subsequent oxidation of cysteines and maintains them in the reduced state. One year later, Grant Fairbanks *et al*. further perfected the protocol for analysis of erythrocyte membrane proteins, by replacing 2-mercaptoethanol with dithiothreitol, a dimercaptan reducing agent more powerful than 2-mercaptoethanol itself [8].

It is common conviction that during electrophoretic separation under the standard conditions described above, proteins are sufficiently well preserved from oxidation, but this turns out to be not true. In fact, the electrophoretic gel is a strong pro-oxidant environment, due to the unavoidable presence of residual traces of ammonium persulfate that is used during the preparation of the gel for catalyzing the polymerization of acrylamide. Furthermore, at the pH value of the electrophoretic gels, both 2-mercaptoethanol and dithiothreitol are in an uncharged state. Therefore they do not migrate together with the proteins and cannot perform their protective function during the electrophoretic run. Generally, this phenomenon occurs during the stacking phase of SDS-PAGE [9], when proteins are highly concentrated into a very small volume. The consequence is the formation of anomalous high-molecular weight protein aggregates that remain at the interface between the stacking and resolving gels.

These artifacts could lead to a misinterpretation of the experimental results. To prevent their onset, the protection of thiol groups by a variety of alkylating agents can be adopted [10]. Alternatively, a simpler method is the treatment of the sample with thioglycolic acid. This compound, owing to its low pKa, is in the anionic state at the pH value of the stacking and resolving gels, and can move towards the anode during electrophoresis. Moreover, the thioglycolate ion is of low molecular weight and migrates more rapidly than all proteins, removing the residual ammonium persulfate before it can react with the proteins themselves [11].

The rate of oxidation depends on the accessibility to oxidants of cysteine residues within the protein, the phenomenon being favored for proteins with high cysteine content. It has been clearly demonstrated, for the human chemokine IP-10, that the elevated propensity to cross-linking mediated by cysteine oxidation during SDS-PAGE can be counteracted by preventive alkylation of cysteines [9]. More recently, evidence for in-gel oxidation has been found also for two different mammalian β-defensins. However, this has not been proven with satisfactory experimental tests [12,13].

The cysteine content of proteins is more variable than that of any other amino acid. Mammalian extracellular proteins have an average cysteine content of 4.1% (as percentage of total amino acids). Conversely, intracellular proteins have an average cysteine content of 1.6% [14]. An intracellular mammalian protein that represents an exception for its high content in cysteines is methionine sulfoxide reductase B1 (MsrB1, originally named SelR), which is one of the 25 selenoproteins of the human genome and one of the few selenoproteins whose function is known [15]. MsrB1 belongs to a large family of enzymes (MsrAs and MsrBs) responsible for the reduction of methionine sulfoxide back to methionine, both free and inserted in polypeptide chains [16]. MsrB1, that is equipped with one catalytic selenocysteine and five cysteines, one of which is involved in the catalytic mechanism together with the selenocyteine residue, has a content of cysteines (including the selenocysteine) of 5.2%, well above the average content of intracellular mammalian proteins.

In **Figure 1**, we disclosed that recombinant human MsrB1 (carrying a cysteine instead of the selenocysteine) when reduced with dithiothreitol and subjected to SDS-PAGE and Western blotting, shows a main band at the apparent molecular mass of 13 kDa. However, other, less intense bands are also visible, which most likely represent protein aggregates with molecular masses of about 25, 40 and 50 kDa (supposedly dimeric, trimeric and tetrameric forms of MsrB1). The use of thioglycolic acid instead of dithiothreitol completely suppresses the formation of these extra bands, clearly indicating that this phenomenon occurs during electrophoresis and involves cysteine oxidation.

MsrB1 belongs to a small group of proteins that show a criticism during electrophoretic analysis due to the extreme reactivity conferred by their high cysteine content. However, it may not be all. Indeed MsrB1, among other MsrB isoforms, exhibits the highest enzymatic activity because of the presence of selenocysteine in its active site. Recombinant MsrB1, in which cysteine was substituted for selenocysteine, displays an 800-fold lower enzymatic activity than the native selenoenzyme [17]. This peculiarity depends on the intense nucleophilicity of selenolate, which is seven orders of magnitude higher than that of thiolate [18]. The elevated chemical reactivity of selenocysteine could render MsrB1 even more susceptible to oxidation during SDS-PAGE.

These uncommon characteristics of MsrB1 are not taken into account in electrophoretic studies of this protein. To the best of our knowledge, only standard SDS-PAGE protocols have been adopted in all publications produced about this topic, including the one published by ourselves concerning Msr enzymes in blood cells [19].

The formation of the described artifacts, not always recognizable *ictu oculi*, could lead to erroneous interpretations of the results. In our opinion, SDS-PAGE separation of proteins with high cysteine content, such as MsrB1, should not be carried out with reducing agents like 2-mercaptoethanol or dithiothreitol, which are unable to guarantee adequate protection. Thioglycolic acid, thanks to the advantageous chemical properties described above, could be the best alternative to ensure the maintenance of protein thiol and selenol groups in their reduced form by preventing oxidation all along the electrophoretic run.

In conclusion, we recommend a careful preventive investigation in order to understand if routinely-employed protocols can be suitable for the study of “special” proteins such as MsrB1. The greater workload that derives from this strategy guarantees a more rigorous approach and could allow the obtainment of more reliable results.

## Figures and Tables

**Figure 1.**
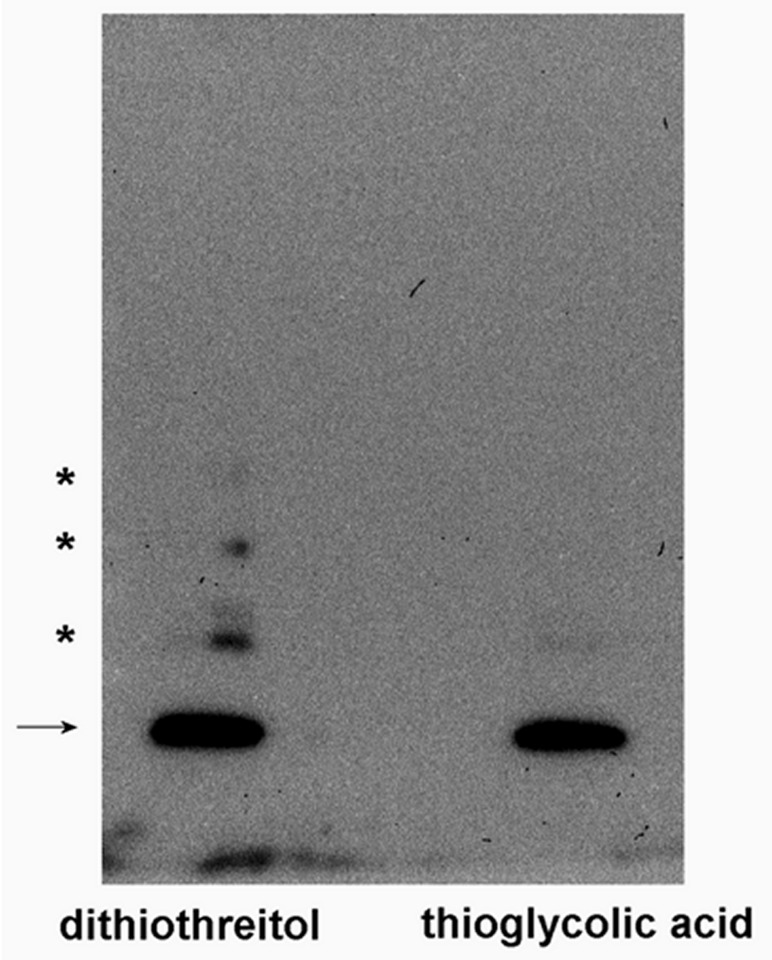
Analysis of recombinant MsrB 1 (Abcam, Cambridge, UK). The procedures were performed essentially as previously reported [19]. MsrB1 was treated with 1% (w/v) dithiothreitol or 0.01% (w/v) thioglycolic acid (final concentrations) and subjected to 5%–15% gradient SDS-PAGE (2 ng of MsrB1 for each sample). After electrophoresis, the proteins were electro-transferred to a PVDF membrane by Western blotting, and then treated with rabbit polyclonal anti-MsrB1 (Abcam, Cambridge, UK) and with the proper secondary horseradish peroxidase-conjugated antibody, for the detection of MsrB1 with the enhanced chemiluminescence detection reagent (GE Healthcare, Chicago, USA). The arrow indicates MsrB1 and the asterisks indicate MsrB1 aggregates.
